# Derivatization of estrogens enhances specificity and sensitivity of analysis of human plasma and serum by liquid chromatography tandem mass spectrometry

**DOI:** 10.1016/j.talanta.2015.12.062

**Published:** 2016-05-01

**Authors:** Abdullah M.M. Faqehi, Diego F. Cobice, Gregorio Naredo, Tracy C.S. Mak, Rita Upreti, Fraser W. Gibb, Geoffrey J. Beckett, Brian R. Walker, Natalie Z.M. Homer, Ruth Andrew

**Affiliations:** a Endocrinology, University/British Heart Foundation Centre for Cardiovascular Science, Queen's Medical Research Institute, University of Edinburgh, 47 Little France Crescent, Edinburgh EH16 4TJ, United Kingdom; b Mass Spectrometry Core, Edinburgh Clinical Research Facility, Queen's Medical Research Institute, 47 Little France Crescent, Edinburgh EH16 4TJ, United Kingdom; c Clinical Biochemistry, Royal Infirmary of Edinburgh, 51 Little France Crescent, Edinburgh EH16 4SA, United Kingdom

**Keywords:** APCI, atmospheric pressure chemical ionization, CID, collision-induced dissociation, cps, counts per second, D, difference, E1, estrone, 3,4-[^13^C_2_E1, 3,4-[^13^C]_2_-estrone, d_4_E1, 2,4,16,16-[^2^H]_4_-estrone, 17αE2, 17α-estradiol, E2, estradiol, ^13^C_2_E2, 3,4-[^13^C]_2_-estradiol, d_4_E2, 2,4,16,16-[^2^H]_4_-estradiol, ESI, electrospray ionization, FA, formic acid, FMP-TS, 2-fluoro-1-methylpyridinium-*p*-toluenesulfonate, HPLC, high performance liquid chromatography, IS, internal standards, LC-MS, liquid chromatography-mass spectrometry, LC-MS/MS, liquid chromatography tandem mass spectrometry, LODs, limits of detection, LOQs, limits of quantitation, MD, mean difference, MRM, multiple reactions monitoring, RME, relative mean error, RSD, relative standard deviation, SD, standard deviation, SNR, signal/noise, SPE, solid phase extraction, TEA, triethylamine, UHPLC, ultra high performance liquid chromatography, Estrone, Estradiol, Derivatization, Liquid chromatography, Mass spectrometry, Methylpyridinium ether

## Abstract

Estrogens circulate at concentrations less than 20 pg/mL in men and postmenopausal women, presenting analytical challenges. Quantitation by immunoassay is unreliable at these low concentrations. Liquid chromatography tandem mass spectrometry (LC–MS/MS) offers greater specificity and sometimes greater sensitivity, but ionization of estrogens is inefficient. Introduction of charged moieties may enhance ionization, but many such derivatives of estrogens generate non-specific product ions originating from the “reagent” group. Therefore an approach generating derivatives with product ions specific to individual estrogens was sought.

Estrogens were extracted from human plasma and serum using solid phase extraction and derivatized using 2-fluoro-1-methylpyridinium-*p*-toluenesulfonate (FMP-TS). Electrospray in positive mode with multiple reaction monitoring using a QTrap 5500 mass spectrometer was used to quantify “FMP” derivatives of estrogens, following LC separation.

Transitions for the FMP derivatives of estrone (E1) and estradiol (E2) were compound specific (*m*/*z* 362→238 and *m*/*z* 364→128, respectively). The limits of detection and quantitation were 0.2 pg on-column and the method was linear from 1–400 pg/sample. Measures of intra- and inter-assay variability, precision and accuracy were acceptable (<20%). The derivatives were stable over 24 h at 10 °C (7–9% degradation). Using this approach, E1 and E2, respectively were detected in human plasma and serum: pre-menopausal female serum (0.5 mL) 135–473, 193–722 pmol/L; male plasma (1 mL) 25–111, 60–180 pmol/L and post-menopausal female plasma (2 mL), 22–78, 29–50 pmol/L.

Thus FMP derivatization, in conjunction with LC–MS/MS, is suitable for quantitative analysis of estrogens in low abundance in plasma and serum, offering advantages in specificity over immunoassay and existing MS techniques.

## Highlights

• Quantitative analysis of low amounts of estrone and estradiol in plasma and serum.
• Quantitation across physiological range in men and pre- and post-menopausal women.
• Methylpyridinium ether derivatives improve analytical specificity and sensitivity.

## Graphical abstract

**Figure:**
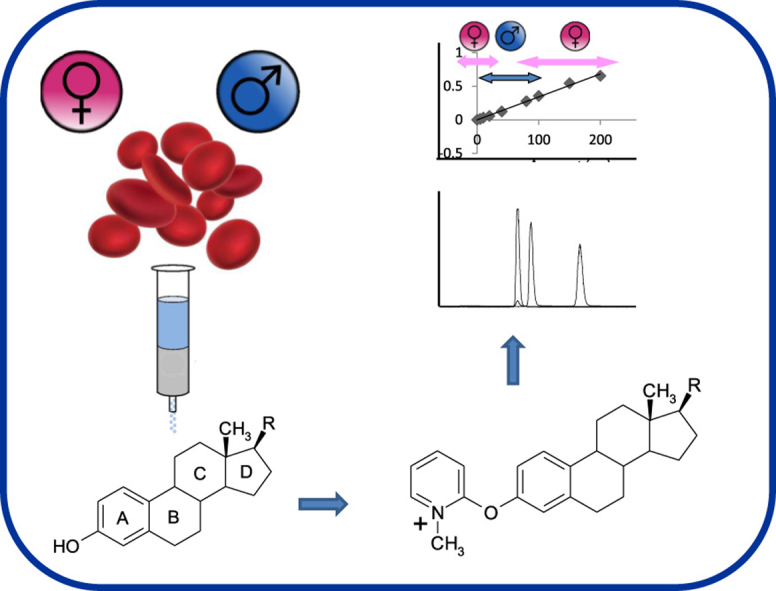

## 1 Introduction

Analysis of endogenous estrogens is challenging due to their extremely low concentrations, e.g. estradiol (E2) can circulate in concentrations less than 20 pg/mL. Concentrations are commonly <50 pg/mL (<184 pmol/L) in men [1] and 2–21 pg/mL (7–77 pmol/L) in postmenopausal women [2]. Moreover their levels reduce with age and in some diseases such as cardiovascular disease [3] and type 2 diabetes mellitus [4]. Although immunoassays have been widely used to quantify across the range of circulating concentrations of estrone (E1; 55–740 pmol/L) [1] and estradiol (E2; 118–915 pmol/L) [5], specificity is challenging due to interference from endogenous isomers and other steroids in the biological matrix [6]. In addition, none of the commercial methods have satisfactory sensitivity and selectivity for use in older female subjects, in whom concentrations are lower [6], [7], [8].

Liquid chromatography–mass spectrometry (LC–MS) provides an alternative analytical method of high specificity and indeed is already replacing immunoassays in the analysis of other steroids routinely monitored in clinical practice, e.g. testosterone and cortisol [6]. However, even with tandem MS coupled to ultra high performance liquid chromatography (UHPLC) [7], [8], estrogens are often present in concentrations below the limit of detection of analysis in human plasma and serum, particularly in samples from post-menopausal women [9].

While ultra-sensitive methods have been developed using state-of-the art tandem MS [10], derivatization improves sensitivity by endowing the analyte with a chargeable or permanently charged group for analysis by MS, changing efficiency of ionization, fragmentation and retention [6], [11]. LC–MS methods have been reported to measure underivatized and derivatized estrogens, with the latter demonstrating improved limits of quantitation (LOQs): for example LOQs of underivatized steroids (E1; 15, E2; 20 pg/mL) [1] versus those of derivatized steroids (E1; 1, E2; 0.5 pg/mL) [2]. Endogenous estrogens have ketone, hydroxyl and phenolic functional groups suitable for targeting by derivatization reagents.

Previous derivatization reagents employed include dansyl chloride [11], [12], [13], [14], [15], [16], pyridine-3-sulfonyl chloride [17], [18], 4-(1H-pyrazol-1-yl)benzenesulfonyl chloride [17], *N*-methyl-nicotinic acid *N*-hydroxysuccinimide ester [19] and isomers of 1,2-dimethylimidazole-sulfonyl chloride [17], [20]. Reaction of the phenolic hydroxyl with a secondary amine moiety of dansyl chloride via nucleophilic aromatic substitution has been the most common approach [11], [12], [13], [14], [15], [16], but a disadvantage for tandem MS is that the product ion is generated by the derivative moiety and hence is not specific for the analyte by mass. In particular the natural mass+2 isotopomers of estrone may generate a background signal within the mass transition of the estradiol derivative [12].

**Fig. 1:**
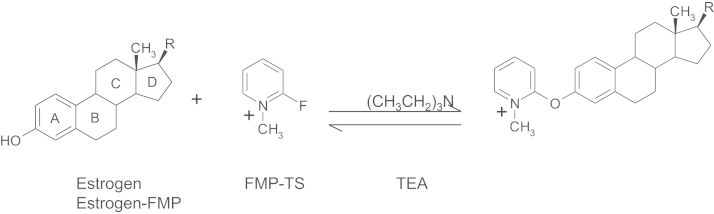
Formation of methylpyridinium ether derivative of phenolic estrogens, showing an example of derivatization of an estrogen with 2-fluoro-1-methylpyridinium*p*-toluenesulfonate (FMP-TS) in the presence of triethylamine (TEA).

Some of these problems can be averted by chromatographic separation but confounding interference can still arise from unknown estrogen metabolites or from stable isotope labeled estrogens used as *in vivo* tracers or internal standards. Therefore a derivative generating analyte-specific precursor and product ions is desirable to improve specificity [6], [13], [17], [20] and permit more rapid chromatography. This may be achieved by using 2-fluoro-1-methylpyridinium-*p*-toluenesulfonate (FMP-TS) (Fig. 1), which has been used previously to detect estrogens in water [13] and human and bovine serum [21].

We aimed to establish an analytical approach using a methylpyridinium derivative of estrogens to detect these hormones in low abundance in human plasma and, in particular, to evaluate if the approach could quantify circulating steroids in post-menopausal females. A highly sensitive method was validated using ion-exchange solid phase extraction, in conjunction with LC–MS/MS.

## 2 Materials and methods

### 2.1 Standards and solvents

Estrone (E1), 17β-estradiol (E2), 17α-estradiol (17αE2), formic acid (FA; ≥98%), triethylamine (TEA; ≥99.5%) and 2-fluoro-1-methylpyridinium-*p*-toluenesulfonate (FMP-TS) were from Sigma-Aldrich, Inc. (St. Louis, USA). 3,4-[^13^C]_2_-estrone (^13^C_2_E1), 2,4,16,16-[^2^H]_4_-estrone (95-97%) (d_4_E1), 3,4-[^13^C]_2_-estradiol (^13^C_2_E2) and 2,4,16,16-[^2^H]_4_-estradiol (d_4_E2) were from Cambridge Isotope laboratories Inc. (Andover, USA). HPLC grade glass distilled solvents (methanol, acetone, hexane and water) were from Fisher Scientific UK Limited (Leicestershire, UK).

### 2.2 Instrumentation

Structural elucidation of derivatives of steroids in solutions of high concentration was performed on a LC triple quadrupole mass spectrometer, a TSQ Quantum Discovery MS (Thermofisher, Waltham, USA) and operated using Xcalibur software version 2.0. Confirmation of accurate mass was performed by direct infusion into a 12 T SolariX dual source Fourier Transform Ion Cyclotron Resonance MS (FT-ICR MS; Bruker Daltonics, MA, US), operated with SolariX control v1.5.0 (build 42.8) software. High-sensitivity quantification was performed on a QTrap 5500 (AB Sciex, Warrington, UK) coupled to an Acquity™ Ultra Performance LC (Waters Corporation, Milford, USA), operated using Analyst software v1.5.1.

### 2.3 Plasma and serum samples

Male and female human plasma and post-menopausal female human serum for method development and validation were from TCS Biosciences (Buckingham, UK), stored at −20 °C. This was prepared from human blood from healthy donors in approved blood collection centers. Plasma was collected into anticoagulant (citrate phosphate dextrose adenine), whereas serum was collected without anticoagulant and allowed to clot naturally.

For method application, serum was collected from 27 pre-menopausal women undergoing investigations for menstrual disorders (24–52 years old); these patient samples were anonymised prior to analysis and ethical approval was thus not required for this method development study. Further plasma samples were obtained from subjects participating in experimental medicine studies for which local ethical approval had been obtained; 20 post-menopausal women (58–60 years old) and 48 men (21–85 years old) men [22].

### 2.4 Standard solutions

Estrogens and internal standards (IS; 5 mg) were dissolved in acetone (5 mL) and stored at −20 °C. Working solutions (1 pg/mL to 100 µg/mL) were prepared by serial dilution on the day of use.

### 2.5 Generation of FMP derivatives

FMP-TS (50 µL; 5 mg/mL in acetonitrile, containing TEA (1%)) was freshly prepared prior to reaction and added to the standard/extract. The mixture was vortexed (10 s) then incubated (40 °C, 15 min). Mobile phase (water: methanol 65:35), containing FA (0.1%, 50 µL) was added to quench the reaction.

### 2.6 Fragmentation analysis of FMP derivatives of estrogens

For product ion characterization by tandem MS at nominal mass, molecular ions of derivatives were isolated by Q1 and subjected to collision-induced dissociation (CID) in Q2 (50 eV E1, 55 eV E2). Q3 was operated in scanning mode from *m/z* 100 to *m/z* 50 above the mass of the molecular ion under analysis. Conditions for multiple reactions monitoring (MRM) were optimized by autotuning during infusion of solutions of the E1 and E2 derivatives (50 ng/mL). Collision energy was optimized to achieve suitable fragmentation for each transition.

For structural elucidation by FTICR-MS, ions of accurate mass (*m/z* 200–1500) were isolated for 20 s prior to CID experiments at 37 eV.

### 2.7 MS tuning of derivatives for quantitiation

MRM conditions for estrogen FMP derivatives were tuned by infusion using a QTrap 5500 mass spectrometer, operated in positive ion electrospray ionization (ESI) mode. Estrogen FMP derivatives (1 µg/mL) were diluted 1:10 in mobile phase (water: methanol 65:35), containing FA (0.1%). Precursor ions for the derivatives were selected; *m/z* 362 for E1 and *m/z* 364 for E2 and 17αE2 (a naturally occurring isomer). Optimized conditions achieving maximal sensitivity for detection of quantifier and qualifier ions are given in Table 1, and were used in conjunction with curtain gas (25 mTorr), collision gas (medium), ion spray (5500 V), temperature (600 °C), ion gas 1 (45) and ion gas 2 (20 mTorr).

### 2.8 Chromatographic conditions

Chromatographic conditions were optimized using an Acquity UPLC® BEH C_18_ column (50×2.1 mm 1.7 µm Waters Corporation, Milford, USA). An isocratic solvent system of water: methanol (65:35), containing FA (0.1%, 0.4 mL/min) was diverted to waste for the initial 2 minutes and maintained for a further 6 min followed by a rapid gradient executed over 2 min until final conditions of water: methanol (5:95), containing FA (0.1%) (0.4 mL/min) were achieved. The column and autosampler temperatures were 25 °C and 10 °C, respectively. Injection volume was usually 20 µL in partial loop mode with needle overfill.

### 2.9 Extraction method

Aliquots of pre-menopausal female serum (0.5 mL), post-menopausal female plasma (2 mL) and male plasma (1 mL) were subject to centrifugation for 20 minutes (8000 g, 4 °C) and sediment removed. The sample volumes were adjusted to 2 mL with water and internal standards (200 pg) added. Solid-phase extraction using Oasis® MCX (3 cc/60 mg, Waters) extraction cartridges was performed under gravity. Prior to loading the sample (2 mL), the cartridges were conditioned with methanol (2 mL), followed by water (2 mL). The sample was loaded and allowed to pass through the cartridges and the eluate discarded. Next, the cartridges were washed with FA (2% v/v, 2 mL) and again the eluate discarded. Finally, the steroids were eluted in methanol (2 mL). Extracts were reduced to dryness under oxygen-free nitrogen (40 °C) and the residues were derivatized as above.

### 2.10 Assay validation

#### 2.10.1 Optimization of derivatization conditions

Reaction conditions were optimized first using aqueous extracts and then extracts of plasma. Conditions evaluated were incubation temperature (25–70 °C), time (5–60 min), reaction volume (25–250 µL) and FMP-TS concentration (0.1–10 mg/mL).

#### 2.10.2 Extraction efficiency

Recoveries of IS from water and plasma were assessed by comparison of signal intensities, following extraction from samples pre- and post-spiked with IS (200 pg; *n*=6). Mean peak areas of derivatives following extraction in pre-spiked samples were divided by those in post-spiked samples and expressed as a percentage.

#### 2.10.3 Assessment of ion suppression

Ion suppression of signal of estrogenic derivatives in the presence of extracts of plasma was evaluated by comparing signal intensity of IS (200 pg) post-spiked into extracted plasma with that of aqueous IS solutions of the same concentration (*n*=6)*.* Mean peak areas of derivatives of steroids from post-spiked extracted plasma was divided by mean peak areas of unextracted standards and expressed as a percentage.

#### 2.10.4 Specificity

Mass chromatograms were inspected at the retention times of analytes and IS for possible interferences by other endogenous compounds in plasma, such as 17αE2. The ratio of quantifier to qualifier ions of each analyte of IS were measured in extracts from plasma and compared with those of standards and accepted if within 20%.

#### 2.10.5 Limit of detection (LOD)

Estrogens (100, 10, 1 ng and 100, 10, 1, 0.1 pg/sample) were analysed by LC–MS/MS and the Signal/Noise (SNR) calculated from peak areas of steroids and adjacent background noise (over the same time window as the peak width). The LOD was assigned as a SNR ~3.

#### 2.10.6 Linearity

Blank samples and aliquots containing estrogens (1, 2.5, 5, 10, 25, 50, 100, 200, 400 pg/sample) and internal standards (200 pg) were analysed by LC–MS/MS. Calibration curves were plotted as the peak area ratio (standard/IS) versus amount of estrogens. Calibration lines of best fit were acceptable if the regression coefficient, *r*, was >0.99. Weightings of 1, 1/*x* and 1/*x*^2^ were compared.

#### 2.10.7 Limit of quantitation (LOQ)

Replicate aliquots (100, 10, 1 ng and 100, 10, 1, 0.1 pg/sample; *n=6*) of estrogens and internal standards were prepared as above and analysed. The LOQ was calculated as that amount affording precision and accuracy of ~20% or less.

#### 2.10.8 Accuracy and precision

Injector reproducibility was tested by injecting the same extracts of male and female plasma 6 times on the same day. The intra- and inter-assay precision and accuracy were assessed using 6 samples (1, 50 and 400 pg/sample) prepared on the same day and different days, respectively, alongside a standard curve (*n*=6). The precision was calculated as the Relative Standard Deviation (RSD) (standard deviation/mean×100), and % accuracy was the Relative Mean Error (RME) ((measured value−theoretical value)×100).

#### 2.10.9 Stability

Stability following storage in the auto-sampler (10 °C) was evaluated by reinjection of a calibration curve and plasma sample after 24 h. The effects of short-term storage in the freezer (−20 and −80 °C) on sample integrity were assessed by reinjection after 24 h and 48 h and 28 days storage.

### 2.11 Method application

Estradiol and estrone were quantified in human serum or plasma from pre and post-menopausal females and males using the validated approach.

## 3 Results and discussion

### 3.1 Method development

#### 3.1.1 Development of derivatization approach

**Fig. 2:**
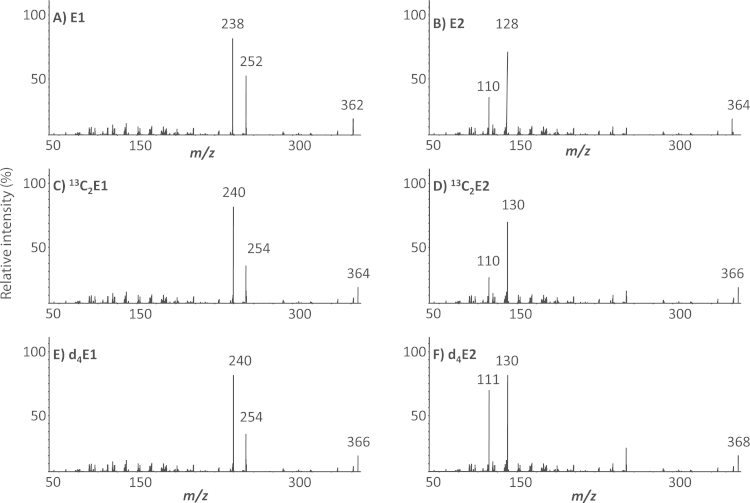
Product ion scans of methylpyridinium ether derivatives of estrogens; (A) estrone (E1) (B) estradiol (E2) (C) 3,4-[^13^C]_2_-estrone (^13^C_2_E1) (D) 3,4-[^13^C]_2_-estradiol (^13^C_2_E2) (E) 2,4,16,16-[^2^H]_4_-estrone (d_4_E1) (F) 2,4,16,16-[^2^H]_4_-estradiol (d_4_E2).

The anticipated reaction is shown in Fig. 1, yielding a positively charged derivative. More intense signals from ions were measured with ESI compared with atmospheric pressure chemical ionization (APCI), typical of species with pre-existing charge. In contrast APCI is often the method of choice for underivatized steroids, due to their low proton/electron capture affinities in liquid-phase [6]. The precursor ions of the derivatives of estrone and estradiol were the expected molecular ions, *m/z* 362 and 364, respectively. However, after CID experiments, product ion spectra of E1 FMP and E2 FMP differed from each other and showed an analyte-specific fragmentation pattern as reported previously [13], [21]. These offer advantages compared with other derivatives such as those formed using dansyl chloride [11], [12], [13], [14], [15], [16]; the product ion (*m/z* 110) representing the derivatization group for both steroids did not dominate (Fig. 2).

**Fig. 3:**
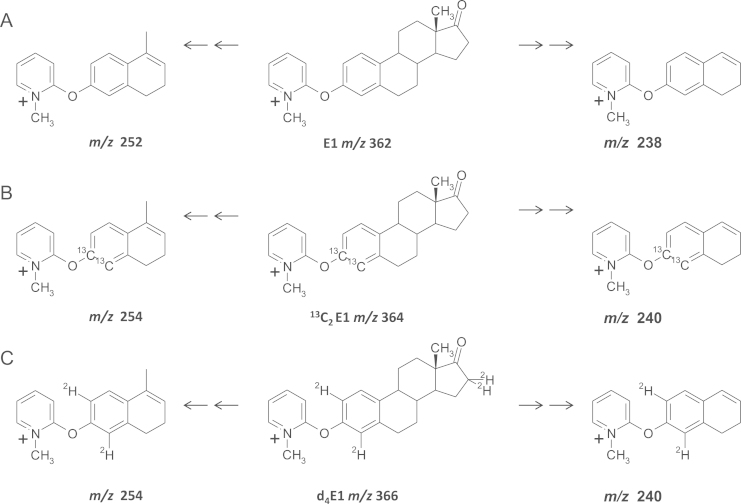
Proposed fragmentation pattern of methylpyridinium ether derivatives of estrogens; (A) estrone (E1) (B) 3,4-[^13^C]_2_-estrone (^13^C_2_E1) (C) 2,4,16,16-[^2^H]_4_-estrone (d_4_E1).

**Fig. 4:**
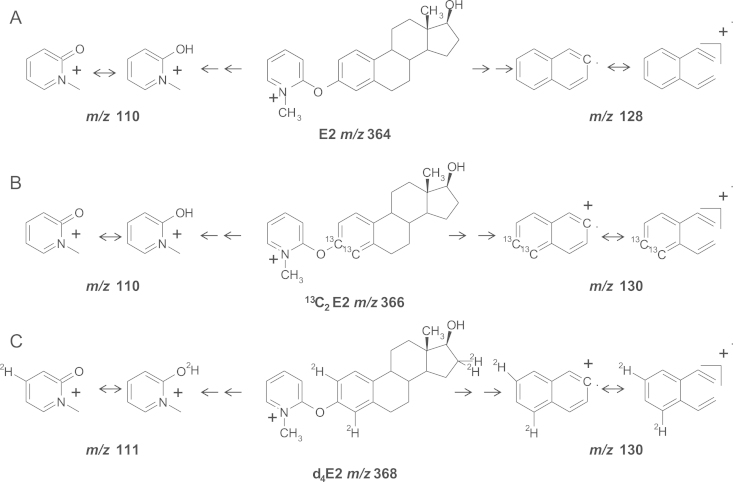
Proposed fragmentation pattern of methylpyridinium ether derivatives of estrogens; (A) estradiol (E2) (B) 3,4-[^13^C]_2_-estradiol (^13^C_2_E2) (C) 2,4,16,16-[^2^H]_4_-estradiol (d_4_E2).

Stable-isotopically labeled estrogens were used to elucidate potential fragmentation pathways and the nature of both quantifier and qualifier ions was proposed, based on a mechanism including α–β cleavages, retro-Diels-Alder, methyl migration and hydrogen shift (McLafferty +1 reaction) [23] (Fig. 3, Fig. 4). High CID at 50 eV of the *m/z* 362 ion formed by E1-FMP generated two product ions of significant abundance at *m/z* 252 and 238. These masses demonstrated an increment by +2 to *m/z* 254 and 240 for both d_4_-E1 and ^13^C_2_-E1, respectively, suggesting the presence of the A ring in the fragment but loss of the steroidal D ring (with 2 deuteriums on C_16_). The formation of *m/z* 252 was proposed to be a charge-remote concerted mechanism involving the elimination of CO as neutral loss before methyl migration from the ^13^C to the C_16_ position in the steroid ring, followed by loss of [C_6_H_10_]^+^ via a four-center concerted mechanism after α-cleavage of the C_14_-C_15_ bond. This may result in potential isomeric structures through the double bond at C_9_. The product ion at *m/z* 238 was proposed to form by loss of CH_2_ (as a radical cation) from C_9_, again creating two possible isomeric structures. FTICR-MS analysis confirmed the structural formulae of the product ions of *m*/*z* 252.13821 and 238.12260 within 0.3 and 0.2 ppm of the theoretical masses (252.13829 and 238.12264 amu; C_17_H_18_ON, C_16_H_16_ON structural formulae, respectively). Similarly for ^13^C_2_-E1, masses of the product ions (*m*/z 254.15090 and 240.13509) were within 0.2 and 0.4 ppm of the theoretical masses (254.15084 and 240.13519 amu; ^13^C_2_-C_15_H_18_ON, ^13^C_2_-C_14_H_16_ON structural formulae, respectively).

Conversely, CID experiments carried out at 55 eV on the FMP-E2 precursor ion at *m/z* 364 generated two product ions of significant abundance but with lower mass, at *m/z* 128 and 110, which underwent a mass increment to *m/z* 130 and 111, respectively for d4-FMP-E2. ^13^C_2_-FMP-E2 in contrast generated product ions of *m/z* 130 and 110. The product ion at *m/z* 110 was assumed to originate from the derivatization tag, proposed to form by β-cleavage of the ether bond at C_4_ followed by a proton migration via a (four or six) center concerted mechanism from either C_2_ or C_4_ of the steroid moiety. Therefore in analysis of d_4_-FMP-E2, an A-ring deuterium may migrate, generating a product ion of *m*/*z* 111. In contrast, the product ion at *m/z* 128 was proposed to form from the steroid moiety, incorporating the A-ring (and hence two deuteriums or two ^13^C atoms). The mechanism may be a charge-remote concerted mechanism causing dehydration of the secondary alcohol at C_17_ with further β-cleavage of the ether bond at C_4_ followed by a retro-Diels-Alder concerted mechanism, resulting in two possible conjugated isomers. Due to the lower masses of the product ions, further confirmation could not be obtained by FTICR-MS. The differences in behavior between the E1-FMP and E2-FMP fragmentation may relate to the unique stabilization of the conjugated system of the E1 related fragment at *m/z* 252, after ketone release and conformational rearrangement.

Accordingly, distinct quantifier ions, derived from the steroidal structure, were selected (Table 1) for E1 and E2 derivatives. Use of *m*/z 128 as the quantifier ion for E2- FMP, as opposed to *m/z* 110, allowed distinction between analyte and A-ring isotopically-labeled estrogens, used either as internal standards or *in vivo* tracers. Phase 1 metabolism of estrogens via oxidation occurs in the A-ring, to form hydroxy and methoxy estrogens on the 2 and 4 positions [9]. Again this derivatization approach would offer greater selectivity against unknown background interferences. Three MS systems were used in this characterization. While they were being applied for different purposes (high versus low concentration analysis and structural elucidation), the fact that the same precursor and product ions could be detected in three MS systems with different source configurations indicated a robust method easily transferable between laboratories. Final tune conditions in ESI mode for the FMP derivatives of estrogens, using the quantifier and qualifier ions selected are shown in Table 1.

#### 3.1.2 Optimization of derivatization reaction conditions

Reaction conditions to generate FMP derivatives were optimized first using aqueous extracts and then extracts of male and female plasma and final conditions reported. The efficiency of reaction was improved by increasing temperature from 25 to 40°C and time from 5 to 15 min, but further improvement was not seen at higher temperatures or with longer incubations. Reassuringly the intensity of signal was not adversely affected upon prolonged incubation, proving robustness of the protocol within day-to-day practice, but was improved by increasing the volume and concentration of FMP-TS from 25 to 50 µL and from 0.1 to 5 mg/mL, respectively

### 3.2 Chromatographic conditions

**Fig. 5:**
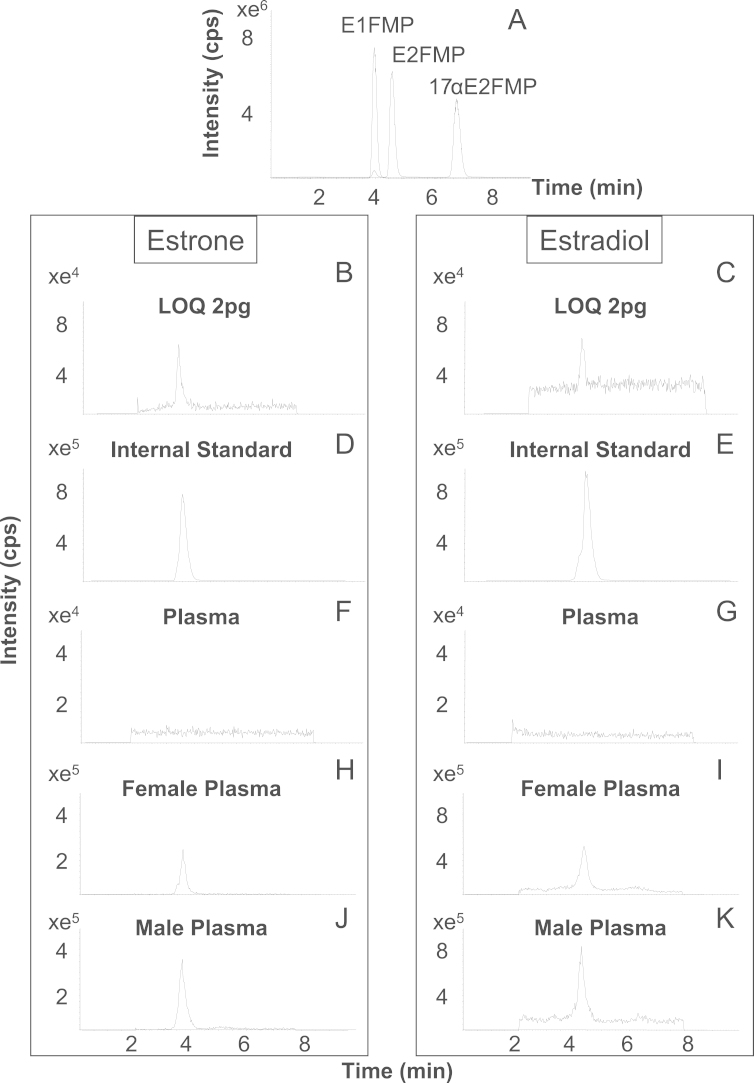
Mass chromatograms of quantifier mass transitions of methylpyridinium ether (FMP) derivatives of estrogens; (A) chromatographic resolution of derivatives of estrone (E1), estradiol (E2) and 17α-estradiol (17αE2) (400 pg) prepared from aqueous standard solutions. Derivatized E1 (B) and E2 (C) from standards prepared with quantities equivalent to the limits of quantitation. Quantifier mass transitions of 3,4-[^13^C]_2_ derivatized estrone (D,F) and 3,4-[^13^C]_2_ -estradiol (E,G) in standards (200 pg) and blank plasma, respectively, demonstrating lack of interferences at the retention times of interest. Derivatized E1 (H, J), E2 (I, K) in extracts of female and male plasma, respectively. Counts per second (cps).

Baseline resolution between estrogen FMP derivatives within reasonable analytical time was achieved using a C18 UHPLC column. Typical retention times for E1, E2 and 17αE2 were 4, 4.5 and 7.5 minutes, respectively (Fig. 5A). At early time points (until 2 minutes) the flow was diverted to waste and towards the end of the analysis where a rapid increasing organic gradient was used; both strategies were employed to minimize build-up of derivatization reagent on the column and in the source and were essential for robustness during larger batch analysis.

### 3.3 Extraction

Previous methods to extract estrogens from biological matrices had often employed solvent extraction, e.g. with methyl *t*-butyl ether [20], and more recently, supported liquid extraction [10]. Following a thorough evaluation, the lipophilic polymeric adsorbent, Oasis®, was ultimately selected for extraction of estrogens [8], [24], [25], [26]; this approach had been used successfully before in monitoring of environmental estrogens. These cartridges showed excellent retention of analytes, and generated a clean extract with reproducible high recovery. Initial development using Oasis® HLB [8], [22], [23], [24] showed excellent recoveries from water (mean±RSD; E1 99±4%, E2 97±2%, ^13^C_2_E1 98±10% and ^13^C_2_E2 96±5%), but significant ion suppression was identified in the plasma extracts (^13^C_2_E1 35%, ^13^C_2_E2 32% versus unextracted standards). Previous problems with ion suppression of derivatized estrogens by phospholipids have been reported [21] and were overcome with online ion-exchange chromatography. Here suppression was minimized by employing weak anion exchange to further clean-up the sample; ultimately use of Oasis® MCX [27] extraction cartridges was validated. Using this approach efficient and consistent recovery of estrogens from plasma was achieved for both analytes (mean±RSD; E1 95±5% and E2 92±3%) and internal standards (^13^C_2_E1 97±12% and ^13^C_2_E 90±6%), with minimum ion suppression (post-spiked vs unextracted; ^13^C_2_ E1 97±7%, ^13^C_2_ E2 93±8%).

### 3.4 Assay validation

#### 3.4.1 Specificity

Baseline chromatographic separation of estrogens (E1 and E2) and endogenous isomers in plasma (17αE2) was achieved (Fig. 5A). In plasma, the ratio of quantifier to qualifier mass transitions was monitored by LC–MS/MS to ensure specificity (E1 1.1, E2 1.1, ^13^C_2_ E1 1.1 and ^13^C_2_ E2 1.4), comparable to those in aqueous solutions, showing differences less than 20% (E1 10%, E2 0%, ^13^C_2_E1 8.3% and ^13^C_2_E2 17.6%). Interferences were not observed in plasma at the retention times of the internal standards (Fig. 5D–G).

#### 3.4.2 Linearity

^13^C_2_ labeled estrogens were selected as internal standards, since loss of deuterium may happen during derivatization, particularly from the A-ring. At the outset of this work only estrogens with two labeled carbons were available, but subsequently those with three have become commercially available and may offer enhanced mass distinction of isotopologues in future studies. Linear standard curves of estrogen FMP derivatives were generated in the range (1–400 pg/sample) with a mean *r* value of 0.9953. Weighting of 1/x^2^ was applied, yielding a mean intercept of 0.0017. This range is similar to that determined using other sensitive derivatives developed recently [18].

#### 3.4.3 Limits of detection and quantitation

The LOD and LOQs of FMP derivatives of both estrogens were 1 pg/sample (~0.2 pg on column) (representative chromatograms of LOQs shown in Fig. 5B and C). This could be achieved using sample volumes of 0.5–2 mL, depending on subject characteristics. This again compares favorably with other recent methods, e.g. 0.5 pg/mL [20], and is an improvement over previous reports using dansyl chloride, albeit with older instrumentation [11]. The larger sized Oasis cartridges were necessary to maintain a clean extract when handling larger volume samples from post-menopausal women.

#### 3.4.4 Precision and accuracy

The values of intra- and inter-assay precision and accuracy were acceptable (<20% RSD for precision and <±20% accuracy) at the LOQ of 1 pg/sample (Table 2) and <15% above this value. Acceptable reproducibility of estrogens upon repeat injection of the same sample was demonstrated with RSD; E1 (1%), E2 (4%) in male and E1 (9%) and E2 (13%) in female plasma.

#### 3.4.5 Stability

The FMP derivatives demonstrated suitable stability upon storage in an auto-sampler (10 °C) over 24 h, with limited degradation measured for derivatized E1 (7%, 5%) and E2 (9%, 6%) in extracts of samples from male and female subjects, respectively. Derivatives were stable upon short-term storage in the freezer (−20 and −80°C, respectively) for 24 h; reduction from original response E1 (2.5%, 1.1%), E2 (3.4%, 1.3%) in male and E1 (2.2%, 1.8%), E2 (3.1%, 1.4%) in female. However, degradation of the derivative was significant after 48 h at −20°C; derivatized E1 (28%, 36%), E2 (29%, 42%) in male and female extracts, respectively. At −80°C, stability of derivatives was acceptable after storage for 28 day; reduction from original response for E1 (8%, 6%), E2 (9%, 8%) in male and female plasma, respectively.

### 3.5 Method application

The method was applied to samples from pre- and post-menopausal females and males and compared to reported biological concentrations (Table 3). In clinical practice immunoassays are used routinely to quantify estradiol, but not estrone, in serum from pre-menopausal women [5], [28]; the LC–MS/MS approach allowed concomitant analysis of E2 and E1 across a wider age range and may be extended to further metabolites.

In pre-menopausal female serum 0.5 mL of serum was required to ensure reliable detection throughout the menstrual cycle. In males, E1 and E2 concentrations were quantified using 1 mL plasma, whereas with plasma from post-menopausal female 2 mL was required to ensure steroids were detected in all subjects. Using these volumes, all concentrations fell within the quantitation limits of the assay of (1–400 pg/sample) for E1 and E2 and quantitiation was achieved within the range of the standard curves.

These volumes and LOQs compared favorably with previous literature. Volumes of serum or plasma from human and bovine in previous MS methods [2], [10], [15], [16], [17], [18], [19], [20], [21] were 100–2000 µL with LOQs 0.1–32 pg on column. For example, Chang et al. [15] used 0.3–1 mL of bovine plasma for simultaneous quantification of E1 and E2, while Yang et al. [19] used 2 mL of human serum to detect estrogens and their metabolites, with LOQs of ~2 pg/on-column. Nelson et al. [16] and Xu et al. [17] reported lower LOQs (~0.3 and 0.2 pg on column, respectively), again requiring between 0.5–2 mL. Wooding achieved similar limits of detection using an approach without derivatisation, but could not robustly quantify estrogens in all post-menopausal females sample tested [10]. Beinhauer et al. [21] has developed a method to trace estrogen FMP derivatives with very low LOQ (~0.7 pg on column) recovered from smaller volumes -100 µL human and bovine serum. However, their approach utilized multiple injection loading with highly specialised equipment. Kushnir et al. [2] likewise quantified dansylated estrogens at even lower LOQs of 0.05–0.1 pg on column, utilizing only 200 µL human serum. However, this approach required the use of 2-dimensional chromatographic separation; again facilities for applying this technique are not commonly available.

## 4 Conclusions

In summary, derivatization to form FMP ethers of phenolic estrogens, in conjunction with LC–MS/MS, is suitable for quantitative analysis of E2 and E1 in low abundance in biological fluids. This approach allows robust measurement across typical physiological ranges found in pre- and post-menopausal women and men, contrasting favorably with immunoassays (for which imprecise measurements are often recorded in the lower physiological range of concentrations [19]). The improvements in sample preparation allowed larger volumes to be processed without significant problems with ion suppression and the use of Oasis® MCX cartridges will likely be compatible with other derivatisation approaches.

Use of FMP-TS reagent offers improved specificity over previously reported derivatization approaches e.g. dansylation, but care should be taken to store derivatized samples at −80 °C if not being analysed within 24 h. Under these conditions all indices of assay quality were acceptable. The derivatization reaction may be applicable to other naturally occurring phenolic steroids.

## Figures and Tables

**Table 1:** Optimized tuning conditions for analysis of methylpyridinium derivatives of estrogens.

**Analyte**	**Precursor ion*****m/z***	**Product ions*****m/z***	**Collision energy (V)**	**Collision cell exit potential (V)**	**De-clustering potential (V)**	**Entrance potential (V)**
***E1***	362	238*	57	10	136	10
252^$^	57	10
***E2***	364	128*	19	18	51	10
110^$^	39	10
^***13***^***C***_***2***_***E1***	364	240*	55	10	61	10
254^$^	57	12
^***13***^***C***_***2***_***E2***	366	130*	63	28	76	10
110^$^	59	12
***d***_***4***_***E1***	366	240*	59	22	81	10
254^$^	57	24
***d***_***4***_***E2***	368	130*	39	14	56	10
111^$^	23	10

Voltage (V); estrone (E1); estradiol (E2); 3,4-[^13^C]_2_-estrone (^13^C_2_E1); 2,4,16,16-[^2^H]_4_-estrone (d_4_E1); 3,4-[^13^C]_2_-estradiol (^13^C_2_E2); 2,4,16,16-[^2^H]_4_-estradiol (d_4_E2); quantifier (*) and qualifier (^$^).

**Table 2:** Precision and accuracy of analysis of estrogens, measured as their methylpyridinium derivatives.

**Precision (%RSD)**	**High (400 pg)**		**Medium (50 pg)**		**Low (1 pg)**	
**Analyte**	**E1**	**E2**	**E1**	**E2**	**E1**	**E2**
**Intra-assay**	3	4	7	10	13	14
**Inter-assay**	5	6	9	15	15	16
**Accuracy (%RME)**						
**Intra-assay**	12	13	12	15	18	17
**Inter-assay**	14	14	14	15	19	19

Estrone (E1) and estradiol (E2). Relative Standard Deviation (RSD) and Relative Mean Error (RME).

**Table 3:** Concentrations of estrogens in males, pre- and post-menopausal females.

**Group**	***N***	**Age (y)**	**pg/mL**		**Reference range (pg/mL)**[15], [26]	
**E1**	**E2**	**E1**	**E2**
**Males**	48	21–85	7–30	16–50	10–60	10–40
**Pre-menopausal females**	27	24–52	36–128	52–196	17–200	15–350
**Post-menopausal females**	20	58–60	6–21	8–14	7–40	<10

Estrone (E1) and estradiol (E2).
